# Four Heat Shock Protein Genes of the Endoparasitoid Wasp, *Cotesia vestalis*, and Their Transcriptional Profiles in Relation to Developmental Stages and Temperature

**DOI:** 10.1371/journal.pone.0059721

**Published:** 2013-03-18

**Authors:** Min Shi, Ya-Na Wang, Ni Zhu, Xue-Xin Chen

**Affiliations:** State Key Laboratory of Rice Biology and Ministry of Agriculture Key Laboratory of Agricultural Entomology, Institute of Insect Sciences, Zhejiang University, Hangzhou, China; University of Geneva, Switzerland

## Abstract

Heat shock proteins (Hsps) play important roles in the environmental adaptation of various organisms. To explore the functions of Hsps in relation to heat stress and development in *Cotesia vestalis*, a solitary larval endoparasitoid of *Plutella xylostella*, four heat shock protein genes, *CvHsp40*, *CvHsc70*, *CvHsp70* and *CvHsp90*, were cloned and sequenced from *C. vestalis* by real-time quantitative PCR and RACE. The cDNA sequence of *CvHsp40*, *CvHsc70*, *CvHsp70* and *CvHsp90* were 1473 bp, 2316 bp, 2279 bp and 2663 bp long, which encode proteins with calculated molecular weights (MW) of 39.1 kDa, 71.2 kDa, 70.1 kDa and 83.3 kDa, respectively. Furthermore, the analysis of genomic DNA confirmed that no introns existed in *CvHsp40, CvHsp70 and CvHsp90* while two introns were present in *CvHsc70*. The amino acid sequence analysis of CvHsps indicated that CvHsp40 is a Type II Hsp40 homolog, CvHsp70 and CvHsc70 are the eukaryotic cytoplasmic Hsp70s, and CvHsp90 is the β-isoform of Hsp90. The divergent transcriptional patterns of *CvHsp40*, *CvHsp70 and CvHsp90* in the different developmental stages suggested that *CvHsp* transcripts were under different mechanisms of regulation during the development of parasitoid larvae. The dramatic increase of transcripts of *CvHsp70* at the third-instar larva coincided with its developmental change in this stage, that is, from inside host to outside host. *CvHsp40*, *CvHsc70* and *CvHsp70* showed a trend of sex-specific differences of transcript abundance in the adult stage. All *CvHsp* transcripts in different developmental stages were significantly induced by heat stress, and the lowest transcript abundances appeared around the temperature 27°C, which probably suggest that this is the most favorable temperature for the development of *C. vestalis*. Our results suggest that the expression of heat shock proteins reflects to some extent the developmental changes and environmental requirements of insects.

## Introduction

The oligophagous solitary larval endoparasitoid, *Cotesia vestalis* (Haliday) ( = *Cotesia plutellae* (Kurdjomov))(Hymenoptera: Braconidae) [1], is one of the major natural enemies of the diamondback moth (DBM), *Plutella xylostella* (L.) (Lepidoptera: Plutellidae), one of the very destructive pests of brassica crops in both small-scale and large-scale farming systems worldwide [2], [3]. *C. vestalis* is distributed in Europe, China, South Africa, Japan, Pakistan, India and Indonesia, and has been introduced from Europe to several countries, including Australia, Commonwealth of Dominica, Fiji, Thailand and the United States and from South Africa to St. Helena [4]. In Hangzhou (China), it is a major parasitoid of *P. xylostella*, and the highest parasitism (57.2%) of DBM recorded [5]. Shi and Liu reported that the optimal survival temperature for *C. vestalis* was 25°C [6]. Above 25°C, the developmental rate increased and the longevity decreased, and no female progeny was produced when the temperature was higher than 35°C.

The heat shock proteins (Hsps) represent a super gene family. On the basis of molecular weight (MW) and homology, Hsps are divided into several families, including Hsp100, Hsp90, Hsp70, Hsp60, Hsp40 and small Hsps (sHsps, the molecular weights ranging from 12 to 43 kDa) [7]–[9]. Hsp40s (also called DnaJs) have been conserved throughout evolution and are important for protein homeostasis, where they stimulate the ATPase activity of the Hsp70s that are involved in protein translation, folding, unfolding, translocation, and degradation [10]. Hsp90s participate in the folding, maintenance of structural integrity, and the proper regulation of a subset of cytosolic proteins, and account for 1% of the soluble protein in most tissues, even in the absence of stress [11].

The Hsps have been widely studied in many fields of biology and a large number of publications describe their molecular and physiological functions, including acting as molecular chaperones that participate in diverse physiological processes including physiological interactions between parasitoid wasps and their host insects [7], [12]–[20]. However, the ecological importance of inducible Hsps has been questioned only recently and was rarely addressed. In the laboratory, it has been shown that very small amounts of induced Hsps from model organisms like *Drosophila melanogaster* can have effects on life history traits such as development, stress resistance, life span and fecundity [21]–[22]. The research and experiments, especially on Hsp70, are processed from laboratory or natural geographic populations of marine organisms, which were exposed to variable environments including occasional stress exposure and environmental conditions. So far, beyond variations in morphology and DNA sequences, variation of temperature tolerance has been accepted as a new bio-indicator of geographic population variations. Meanwhile, the transcript abundances of *Hsp* genes provide a link between variation of temperature tolerance and geographic populations.

In the present study, we explore three issues. First, we identify the sequences of four *C. vestalis Hsp* genes. Second, we describe how the transcript abundances of *C. vestalis Hsp* genes vary during development. Third, we show that the transcript abundances of *C. vestalis Hsp* genes reflect temperature adaptations of local populations.

## Materials and Methods

### Insects and thermal treatments

Pupae and parasitized larvae of *P. xylostella* by the endoparasitoid *C. vestalis* were initially collected from cabbage fields in the suburbs of Hangzhou, Zhejiang province, China. Once emerged, both *P. xylostella* and *C. vestalis* were raised on cabbage grown at 24°C, 65% relative humidity, and 14 h light: 10 h dark. Adult wasps were fed with 20% (v/v) honey solution and propagated using *P. xylostella* larvae as hosts.

*C. vestalis* larvae undergo 3 instars before pupation, and are physiologically staged using previously established morphological criteria [23]. Briefly, the first and second larval instars molted inside the host, and the third instar emerged from the host to spin a cocoon; each instar lasted 2, 5, and 1 day, respectively; the adults emerged at 5 days after pupation.

For thermal treatments (24°C -control, 27°C, 32°C, 37°C and 42°C), groups of 15 first-instar larvae, early second-instar larvae and later second-instar larvae, all developing in host larvae, third-instar larvae, pupae and new emerged (one-day-old) adults were collected into 10 ml cotton-plugged tubes in a glycerol bath (Programmable Temperature Controller DFY-5/10, Nanjing Keer Biotechnology Ltd, Nanjing, China) and set at a selected temperature for 1 h. After thermal treatment, all the treated larvae, pupae and adults were flash-frozen in liquid nitrogen and stored at −70°C until RNA exaction. Each treatment with 15 individuals was replicated 3 times.

### Total RNA and Genomic DNA isolation, cDNA Synthesis and Cloning of CvHsps

One-day-old female adults were processed for cDNA cloning. Total RNA was isolated using TRIzol reagent (Invitrogen, Carlsbad, CA). Residual genomic DNA was removed using RNase-free DNase I (Promega, Germany), and 2 μg RNA was used to generate the cDNAs with a RevertAidTM First Strand cDNA Synthesis Kit (Fermentas, Lithuania). The cDNA fragments of *CvHsp40*, *CvHsp70*, *CvHsc70* and *CvHsp90* were obtained by degenerate primers (Table 1) based on the conserved nucleotide sequences of insects which were deposited in GenBank. The gene specific primers of *CvHsp40*, *CvHsp70*, *CvHsc70* and *CvHsp90* (Table 1) were designed for amplifying the full cDNA sequences using a 5′-Full Race Kit and 3′- Full Race Kit (TaKaRa, Dalian, China) and the full open reading frame (ORF) sequences of *CvHsp40*, *CvHsp70*, *CvHsc70* and *CvHsp90* were verified by PCR. Adult wasp genomic DNA was isolated using the DNeasy Tissue Kit (Qiagen, Germany), and the introns of *CvHsc70* were amplified using ORF-verified primers (Table 1).

**Table 1:** Sequences of Primers.

Gene	Direction 5′→3′	*Sequence*	Used for
Cvhsp40	forward	GCNGARGCNTAYGANGTGCT	degenerate PCR
	Reward	TTBGTDCCNKCCTTCCAKCC	degenerate PCR
	outer primer	AGGCGATCAAGGTCGTGGTA	3′RACE
	inner primer	ACCATTCCCGAAAGAACCATCA	3′RACE
	outer primer	ATGGTTCTTTCGGGAATGGT	5′RACE
	inner primer	CCACGACCTTGATCGCCTTCTT	5′RACE
	forward	ATGGGTAAAGACTACTATAAAACTCTTGGG	verified ORF
	Reward	TCAATTAGGTAGAGTGTCATACAGTATGTCTT	verified ORF
	forward	CGGTGGTGCTGAAACATA	real-time qPCR
	Reward	GGTGGGTCTTGAGCGTGA	real-time qPCR
Cvhsp70	forward	ACWGTWCCXGCTTAYTTCAA	degenerate PCR
	Reward	ACATCRAAGGTDCCRCCGCC	degenerate PCR
	outer primer	TGCCAGCATACTTCAACGATTC	3′RACE
	inner primer	TGCGATTGCTGGGCTGAACG	3′RACE
	outer primer	CGATACCCAGAGATAGGGGAGCAAC	5′RACE
	inner primer	GGATGCGAGTAGAACCTCCCACGA	5′RACE
	forward	ATGCCTGCCATTGGTATT	verified ORF
	Reward	TTAGTCAACTTCTTCAACCGT	verified ORF
	forward	GTGGGAGTGTGGCAACAAGGG	real-time qPCR
	Reward	GTGTCCGTGAAGGCAACATAGC	real-time qPCR
Cvhsc70	outer primer	GTCCCTTGTCGTTGGTGATGGTG	5′RACE
	inner primer	CGTAGGTGGTGAAGGTCTGGGTT	5′RACE
	forward	AAATGACGAAAGCACCCGC	verified ORF
	Reward	ACCTGAATAGGCAGTGGAGTGAC	verified ORF
	forward	TTGATTTGGGAACTACATAC	real-time qPCR
	Reward	AGTCGCTCAGTGTCTGTAAA	real-time qPCR
Cvhsp90	forward	GCKGAGATCGCYCAGCTKATGTC	degenerate PCR
	Reward	GCCTTCATGATRCGYTCCATGTTGGC	degenerate PCR
	outer primer	CGTGAGGAAGACAAAGCCAAAT	3′RACE
	inner primer	CCCTGCTGTATCGTTACTTCTC	3′RACE
	outer primer	TTGGCTTTGTCTTCCTCACG	5′RACE
	inner primer	CGATACAGCAGGGCGAGT	5′RACE
	forward	ATGCCGGAAGGAATGGATACCT	verified ORF
	Reward	TTAATCGACTTCTTCCATACGAGACG	verified ORF
	forward	CTCGCCCTGCTGTATCGT	real-time qPCR
	Reward	ATCGTCAAGTGAGAACCC	real-time qPCR
Cv18SrRNA	forward	CGCCTTTCAAGATACCAAAATACGCC	real-time qPCR
	Reward	TAGCTCTTTCTTGATTCGGTGGGTG	real-time qPCR

Amplified fragments were purified using the QIAquick Gel Extraction Kit (Qiagen, Germany) and ligated directly into the pGEM-T Cloning Vector (Promega, Madison, WI). Each fragment-containing plasmid was isolated from cultured *E. coli* cells by an alkaline miniprep method. Insert fragments were verified by PCR using M13 forward and reverse primers. Sequencing was conducted on an automated fluorescence sequencing system ABI3730 (Applied BioSystems, Foster, CA).

### Sequence analysis

Nucleotides and deduced amino acid sequences were analyzed using DNASTAR programs (Version 5.02) (DNASTAR, Inc., Madison, WI, USA). The functional domains and motifs of *CvHsps* were identified using the programs ScanProsite, Motifscan and SignalP4.0 online (http://www.ca.expasy.org). The obtained amino acid sequences of CvHsps were used to search for homologs in GenBank by BLAST (Position-Specific Iterated-BLAST) software available at the NCBI website (http://www.ncbi.nlm.nih.gov/blast/Blast.cgi). The sequence alignment was performed with Clustal X version 1.81 using default parameters [24] and edited by GeneDoc (Version 2.04) (Free Software Foundation, Inc., MA, USA). The Maximum parsimony (MP) method was used for phylogenetic analysis with MEGA 5.1 [25], and bootstrap analysis provided support values for the branches [26].

### Real-time qPCRs

Real-time qPCR was performed to further compare expression levels of *CvHsp40*, *CvHsp70*, *CvHsc70* and *CvHsp90* in *C. vestalis.* Total RNA was extracted from whole insect bodies by using the TRIzol reagent (Invitrogen, Carlsbad, CA) and was further cleaned by using an RNeasy MiniElute Cleanup kit (Qiagen). The quality and concentration of the RNA was determined using a NanoDrop ND1000 spectrophotometer (NanoDrop Technologies, Roackland, DE, USA). Total RNA from each developmental stage and thermal treatment was checked for genomic DNA contamination by PCR amplification of each RNA sample using ORF verified primers for *CvHsc70*. The amplified products and the DNA ladder were analyzed on a 2% agarose gel containing Ethidium Bromide (EB).

Real-time qPCR reactions were run on an Eco^TM^ Thermal Cycler (Illumina) in 10-μl reactions. Each 10 μl reaction contained 1 μl template cDNA, 5 μl Thunderbird Sybr qPCR Mix (TOYOBO, Osaka, Japan), 1 μl each of the corresponding forward and reverse primers (4 μM) and 2 μl ddH2O. Primer pairs used for real-time qPCR experiments were designed from ORF sequences of *CvHsps* (Table 1). To normalize differences in total RNA amounts that were reverse-transcribed and added to each reaction, *18S rRNA* from *C. vestalis* (*Cv18SrRNA*) (GenBank accession No. JX399880) was used as an active endogenous control. Based on T_m_ value of primer pairs, cycling conditions were designed as: 1 min initial denaturation step at 95°C, followed by 40 cycles of 15 s denaturation at 95°C, 35 s annealing at 60°C, then one cycle of 15 s at 95°C, 15 s at 60°C, and 15 s at 95°C in order to produce the melting curves data. Data were acquired during the extension step and analyzed with the Eco^TM^ Real-Time PCR Detection System. Each amplification reaction was carried out in three biological replicates, from which mean threshold cycle (C_T_) values plus standard deviations were calculated. The plasmid pGEM-T, which contained full ORF sequences of *CvHsp genes* or a 450 bp fragment of *Cv18SrRNA*, was diluted 10-fold in PBS buffer with 10^5^ to 10^1^ copies per reaction. Amplification efficiencies (E) of semi-quantitative real-time qPCRs were determined based on slope values obtained from linear regressions, where C_t_ values were plotted versus the logarithmic values of serially diluted input plasmid DNA templates by employing the equation E = 10^(−1/Slope)^-1 [27]. Here, amplification efficiencies (E) of Cv*Hsp40*, *CvHsp70*, *CvHsc70*, *CvHsp90* and *Cv18SrRNA* were 104.2%, 103.2%, 94.7%, 97.1% and 98.7%, respectively.

Relative transcript amounts of *CvHsps* for each developmental stage and different temperature stresses were determined using the comparative C_t_ method [28]. First, we normalized the C_t_ values for differences in the quantity of cDNA in each reaction by subtracting the observed C_t_ values from our internal control, *Cv18SrRNA*, to generate ?C_t_ values. Then, we confirmed that the C_t_ values of the internal control did not differ between developmental stages (ANOVA, d*f*  = 6, F = 0.655, p = 0.687) or different thermal stress temperatures (one day old female adults, ANOVA, df  = 4, F = 0.311, p = 0.864).

### Statistical analysis

The relative transcript amounts of *CvHsps* were analyzed using one-way analysis of variance (ANOVA). The differences in relative transcript amounts of *CvHsps* were compared using Dunnett's multiple comparison and LSD comparison post hoc tests. All statistics were performed using the SPSS software (SPSS 16.0, SPSS Inc., Chicago, IL).

## Results

### Sequence analysis of the CvHsps

#### CvHsp40

**Figure 1:**
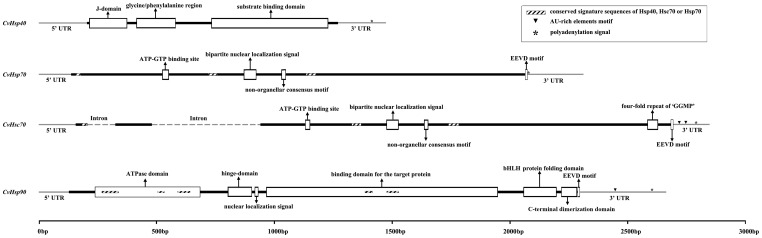
Schematic representation of the full cDNAs for *CvHsp40*, *CvHsc70*, *CvHsp70* and *CvHsp90* of *Cotesia vestalis*.

The full length *CvHsp40* cDNA (GenBank accession no. JX088376) contains an ORF of 1068 bp encoding a 355 amino acid protein with a predicted molecular weight of 39.1 kDa and theoretical isoelectric point (p*I*) of 9.12 (Fig. 1 and Fig. S1). Three conserved regions are found in the deduced amino acid sequence of CvHsp40. The first one is a N-terminal J-domain, located at aa 3-57. The second is a glycine/phenylalanine region (G/F domain, aa 70–125). The last region is a C-terminal substrate binding domain (C domain, aa 176–341). Comparing the cDNA and genomic sequences revealed no intron in *CvHsp40*.

#### Cvhsp70

The full length *CvHsp70* cDNA (GenBank accession no. JX088377) contains an ORF of 1938 bp encoding a 645 amino acid protein with a molecular weight of 70.1 kDa and theoretical p*I* of 5.35 (Fig. 1 and Fig. S2). By Motifscan analysis, we found three conserved characteristic signatures, including IDLGTTYS (aa 6–13), IFDLGGGTFDVSIL (aa 194–207) and VVLVGGSTRIPKIQS (aa 332–346), and four motifs, including an ATP-GTP binding site AEAYLGQK (aa 130-137), a bipartite nuclear localization signal sequence (NLS) ERKYRKNLKTNPRALRRL (aa 244-261), a non-organellar consensus motif RARFEEL (aa 297–303) and a cytoplasmic characteristic motif EEVD (aa 642–645). Comparing the cDNA and genomic sequences revealed no intron in *CvHsp70*.

#### CvHsc70

The full length *CvHsc70* cDNA (GenBank accession no. JX088378) contains an ORF of 1956 bp encoding a 651 amino acid protein with a predicted molecular weight of 71.2 kDa and a theoretical p*I* of 5.26 (Fig. 1 and Fig. S3). Additionally, there are two AU-rich elements (ARE; AUUUA motif) located at 25–29 nt and 72–76 nt downstream of the termination codon in the 3′ UTR. By Motifscan analysis, we found three conserved characteristic signatures, including IDLGTTYS (aa 9–16), IFDLGGGTFDVSIL (aa 197–210) and VVLVGGSTRIPKIQS (a 334–348), and five motifs, including an ATP-GTP binding site AEAYLGQK (aa 131–138), a bipartite nuclear localization signal sequence (NLS) KRKYKKDLTSNKRAERRL (aa 246–263), a non-organellar consensus motif RARFEEL (aa 299–305), a four-fold repeat of the tetrapeptide “GGMP” (aa 615–630) and a cytoplasmic characteristic motif EEVD (aa 648–651). The sequence of the *Cvhsc70* gene contains 2 introns of 119 and 460 bp length.

#### CvHsp90

The full length *CvHsp90* cDNA (GenBank accession no. JX088379) contains an ORF of 2172 bp encoding a 723 amino acid protein with a predicted molecular weight of 83.3 kDa and a theoretical p*I* of 4.996 (Fig. 1 and Fig. S4). By Motifscan analysis, we found all five highly conserved signature sequences defining the Hsp90 family of known eukaryotes, NKEIFLRELISNSSDALDKIR (aa 35–55), LGTIAKSGT (aa 102–110), IGQFGVGFYSAYLVAD (aa 126–141), IKLYVRRVFI (aa 351–360) and GVVDSEDLPLNISRE (aa 377–391), as well as a consensus sequence MEEVD at the C-terminus. We also found: (a) a typical histidine kinase-like ATPase domain (aa 37–186) which is ubiquitous in all Hsp90 family members; (b) two highly charged domains, one a hinge-domain (aa 225–259) and the other a C-terminal domain (aa 691–716); (c) a nuclear localization signal (KKKKKK) (aa 263–268); (d) the binding domain for the target protein(s) (aa 279–607) and a basic Helix-Loop-Helix (bHLH) protein folding domain EADKNDKSVKDLVVLLFETALLSSGFSLDDPQVHAARIYRMIKLGLGI (aa 643–690). Comparing the cDNA and genomic sequences revealed no intron in *CvHsp90*.

**Figure 2:**
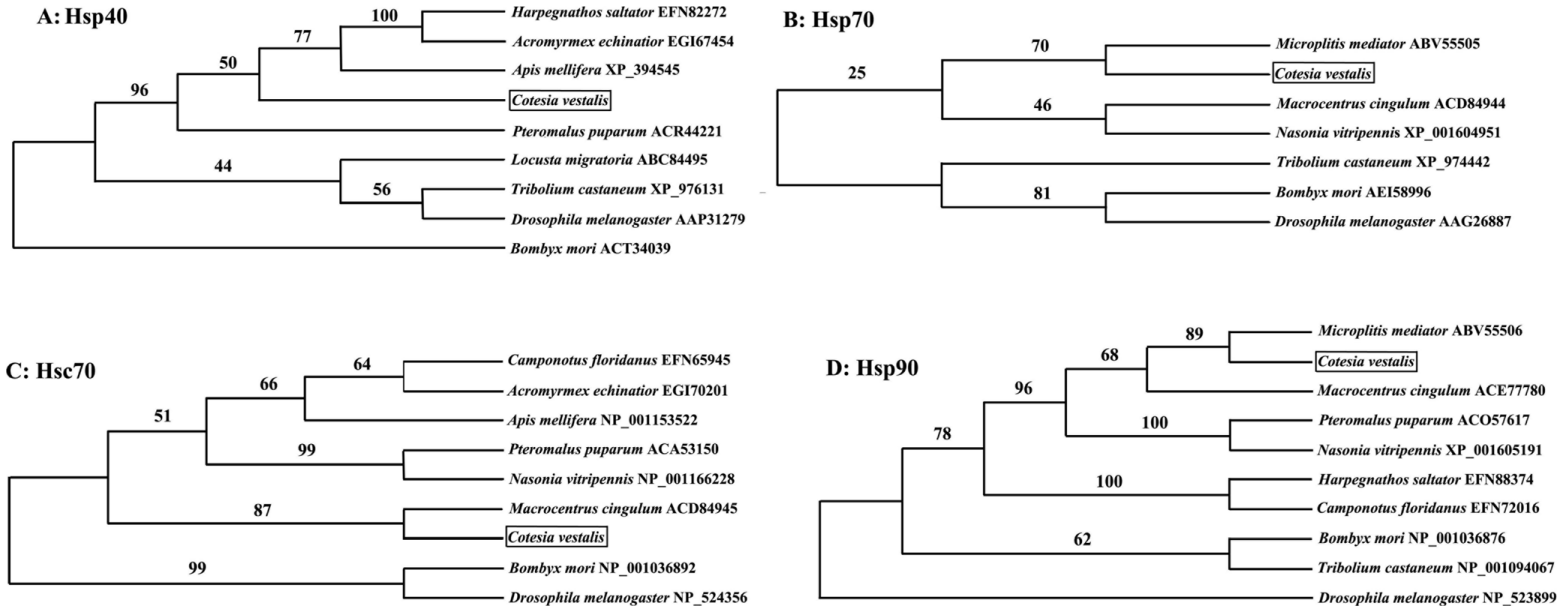
Phylogenetic analysis of CvHsps and other correspondence homologs from Hymenoptera. The Maximum Parsimony (MP) tree is generated from MEGA 5.01, and the numbers on the branch are the bootstrapping values. The positions of Hsps of *Cotesia vestalis* are boxed.

Homologs of CvHsps were found among hymenopteran species by PSI-BLASTP. By using the Parsimony method of tree reconstruction, we revealed that the most phylogenetic closed homolog group of CvHsp40 was (AmHsp40+ (HsHsp40+ AeHsp40)), and they shared identity of 77–79% (Fig. 2A). Meanwhile, the most phylogenetic closed homologs of CvHsp70, CvHsc70 and CvHsp90 were MmHsp70, McHsc70 and MmHsp90, respectively, and they shared identity of 94%, 95% and 86%, respectively (Fig. 2B–D).

### Transcriptional profiles of CvHsps during different developmental stages

**Figure 3:**
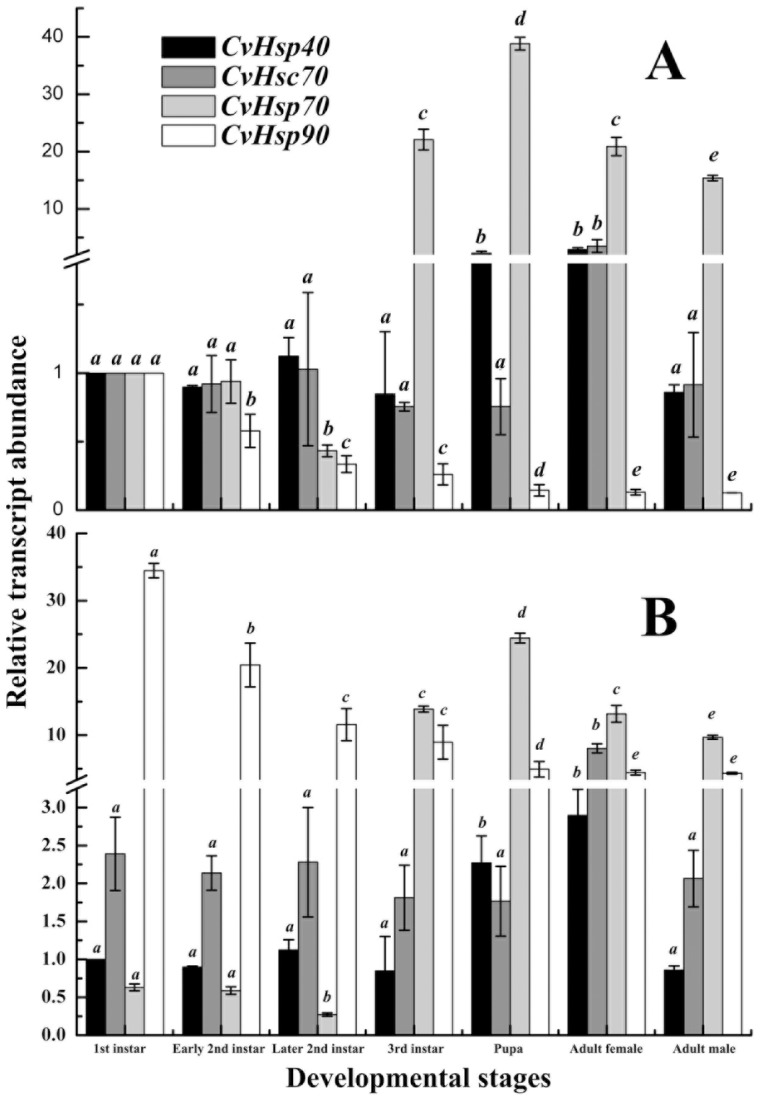
Relative transcript abundances of *CvHsps* during developmental stages at 24°C. The quantity of each *CvHsps* mRNA was normalized to the abundance of *Cv18SrRNA*. Subsequently, the normalized value of each *CvHsps* was divided by the mount of the corresponding *CvHsp* of first-instar larva (A) or by the mount of *CvHsp40* of first-instar larva (B). Columns topped by different letters indicate significantly different means within the relative transcript abundances of a given *CvHsp* gene at different developmental stages by ANOVA analysis (p<0.05).

To profile the transcriptional pattern of *CvHsps* during development at 24°C, mRNA levels of the four *CvHsps* were analyzed at different developmental stages, including first-instar, early second-instar, later second-instar, and third-instar larvae, pupae, female and male adults. First, the quantity of each *CvHsp* mRNA was normalized to the abundance of *Cv18SrRNA*. Then, this normalized value was divided by the amount of the corresponding *CvHsp* of first-instar larva, and the fold difference was used in the analyses of the relative transcriptional levels of the corresponding *CvHsp* during the development (Figure 3A).

The transcriptional level of *CvHsp40* was almost the same throughout the larval stage, but increased significantly at pupal and adult stages (female). The transcriptional level of *CvHsc70* was similar during the larval, pupal and male adult stages. The transcriptional level of *CvHsp70* was generally low (Fig. 3B) and slightly decreased in early and middle larval stage, including first-instar, early second-instar and later second-instar larval stages, but dramatically increased at the following third-instar larval stage and reached its peak at pupal stage, and then decreased again in adult stages. The transcriptional level of *CvHsp90* was highest at the first-instar larval stage, and then dropped approximately 7 folds to a relatively low level at later developmental stages. The transcripts of *CvHsp40*, *CvHsc70* and *CvHsp70* in female adult were all significantly more abundant than those in male adult, however the transcript abundance of *CvHsp90* in female adult was quite close to that in male adult.

We also tried to compare the transcript abundance within four *CvHsps* at a given developmental stage. Therefore, the normalized value by the abundance of *Cv18SrRNA* was then divided by the amount of *CvHsp40* of first-instar larva (Figure 3B). We found that *CvHsp70* had the lowest transcript abundance in early and middle larval stages while *CvHsp90* had its highest transcript abundance. However, in third-instar larval and following developmental stages, *CvHsp70* had the highest transcript abundance.

### Transcriptional profiles of CvHsps after thermal treatments

**Figure 4:**
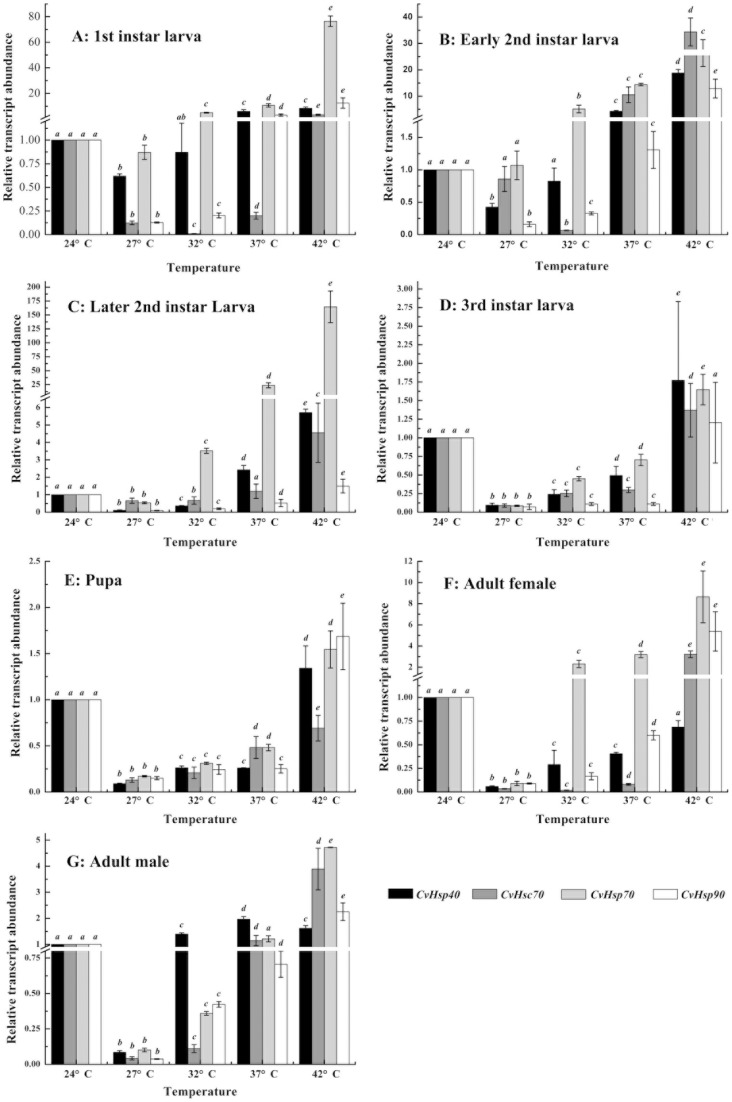
Relative transcript abundances of *CvHsps* of different developmental stage under thermal stress. The quantity of *CvHsp* mRNA is normalized to the abundance of *Cv18SrRNA*. Subsequently, the normalized value of each *CvHsp* is divided by the amount of the corresponding *CvHsp* at 24°C of each developmental stage, respectively. Columns topped by different letters indicate significantly different means within the relative transcript abundances of a given *CvHsp* gene under different temperatures by ANOVA analysis (p<0.05). A-G represents first-instar larva, early second-instar larva, later second-instar larva, third-instar larva, pupa, female adult and male adult, respectively.

**Figure 5:**
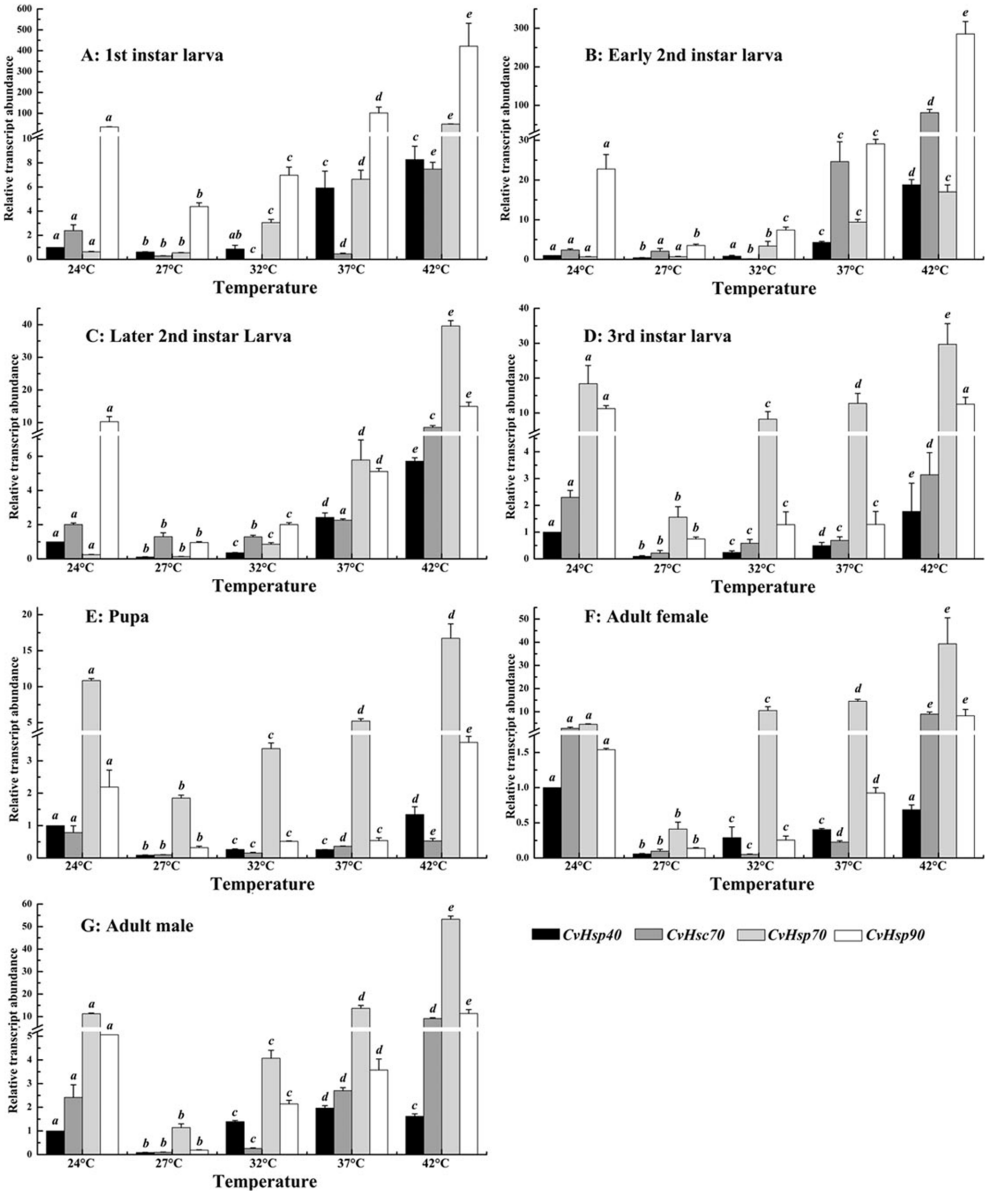
Relative transcript abundances of *CvHsps* of each developmental stage under thermal stress. The quantity of *CvHsp* mRNA is normalized to the abundance of *Cv18SrRNA*. Subsequently, the normalized value of each *CvHsp* is divided by the mount of *Cvhsp40* of the corresponding developmental stage at 24°C. Columns topped by different letters indicate significantly different means within the relative transcript abundances of a given *CvHsp* gene under different temperatures by ANOVA analysis (p<0.05). A-G represents first-instar larva, early second-instar larva, later second-instar larva, third-instar larva, pupa, female adult and male adult, respectively.

To profile the transcriptional pattern of *CvHsps* under different temperatures (24°C, 27°C, 32°C, 37°C and 42°C), mRNA levels of the four *CvHsps* were analyzed at different developmental stages, including all the larval stage, pupae, and female and male adults. First, the quantity of each *CvHsps* mRNA was normalized to the abundance of *Cv18SrRNA*. Then, the normalized value of each Cv*Hsps* was divided by the amount of the corresponding *CvHsp* at 24°C of each developmental stage, respectively, and the fold difference was then used in the analyses of the relative transcriptional levels of a given *CvHsp* at different temperatures (Fig. 4). To further compare the transcript abundance within four *CvHsps* of a given developmental stage at different heat temperatures, the normalized value of each Cv*Hsps* was again divided by the amount of *CvHsp40* at 24°C of the corresponding developmental stage (Fig. 5).

The transcriptional pattern of four *CvHsp*s indicated that 27°C was a “turn-over” temperature, where the transcriptional levels of *CvHsp40*, *CvHsc70*, *CvHsp70* and *CvHsp90* at different developmental stages were significantly lower than those at 24°C, 32°C, 37°C and 42°C, except for the lowest transcript abundance of *CvHsc70* of first-instar larva, early-instar larva and female adult were appear at 32°C. When the temperature was higher than 27°C, and 32°C for *CvHsc70* of above developmental stages, the transcriptional levels of *CvHsp40*, *CvHsc70*, *CvHsp70* and *CvHsp90* of each developmental stage were increased obviously in response to thermal stress (Fig. 4), suggesting that the transcripts of *CvHsp40*, *CvHsc70*, *CvHsp70* and *CvHsp90* were significantly induced by heat stress. Here, we also noticed that the transcriptional peak of *CvHsp40* in male in response to different heat stress showed at 37°C not 42°C (Fig. 4G). *CvHsp90* had the highest transcriptional level at first- and early second-instar larvae (Figure 5A–B) while *CvHsp70* had its highest transcriptional level at third-instar larval, pupal and adult stages (Figure 5D–G) among four tested *CvHsps* under all test temperatures. However, there was no such clear transcriptional pattern of *CvHsp70* or *CvHsp90* in later second-instar larva (Figure 5C). Additionally, the sensitivities of *CvHsp40*, *CvHsc70*, *CvHsp70* and *CvHsp90* to the heat treatment during different developmental stages were different from each other. When the temperatures increased from 27°C to 42°C, the transcriptional levels of four *CvHsp*s were all up-regulated at least 5 folds during larval and pupal stages (Fig. 4A–E), but displaying irregular patterns of heat sensitivity. When comparing the up-regulated ratios of the transcript abundances of *CvHsp40*, *CvHsc70*, *CvHsp70* or *CvHsp90* between female and male adults (Fig. 4 F–G), *CvHsp70* and *CvHsc70* were greater in females (91.9 folds and 93.9 folds) than in males (47 folds and 69.4 folds) while *CvHsp40* was smaller in females (11.7 folds) than in males (18.9 folds), but *CvHsp90* exhibited no differences between males and females.

## Discussion

Heat shock proteins are key elements of the stress response system at the cellular level in all organisms. They are up-regulated in cells exposed to a wide variety of abiotic stressors, such as heat shock, osmotic stress, and environmental contaminants (heavy metals, pesticides and polycyclic aromatic hydrocarbons), and biotic (bacteria and virus) factors [9]. In the present study, using RACE or direct PCR with primers designed on the basis of conserved Hsp genes sequences, we identified four genes encoding Hsps, including *CvHsp90*, *CvHsp70*, *CvHsc70* and *CvHsp40*, in *C. vestalis*. The predicted amino acid sequences of these proteins showed high similarity to Hsp sequences known from other Hymenoptera, with identity in the range of 76–86% for CvHsp90, 89–94% for CvHsp70, 92–95% for CvHsc70 and 77–79% for CvHsp40. These similarities add confidence to our identifications of genes encoding HSPs in a parasitoid wasp.

Amino acid sequence comparisons revealed that all core signatures or motifs were characterized in these Hsps. We identified five signatures for CvHsp90, three for CvHsp70 and CvHsc70, and two for CvHsp40, plus other motifs. None of the four conserved repeats with the consensus sequence CxxCxGxG (cysteine-rich region or zinc finger motif) was found in the amino acid sequence of CvHsp40, which indicated that it was the Type II Hsp40s [29]. Compared with Type I Hsp40, Type II Hsp40s also can form chaperone pairs with cytosolic Hsp70 and help folding proteins but with much lower efficiency [30]. The well conserved C-terminal motif MEEVD or EEVD argue that these motifs enable CvHsp90, CvHsp70 or CvHsc70 to bind other co-chaperones [31], which also indicated that CvHsp90, CvHsp70 and CvHsc70 are cytosolic Hsps [32]. The non-organellar stress protein motif “RARFEEL” and bipartite nuclear localization signal “(K/R)2(X)nRRLRT” motif suggest that CvHsp70 and CvHsc70 not only belong to the eukaryotic cytosolic-cytoplasmic Hsp70 family but also can selectively translocate into the nucleus of cells [33]. Comparing CvHsp70 and CvHsc70, no “GGXP” motif occurs near the 3′- terminal of CvHsp70, whereas CvHsc70 contains four “GGXP” repeats, which suggests CvHsc70 has a stronger binding affinity in co-chaperone binding activities [34]. There was no glutamine-rich sequence (QTQDQ) be found located at the N-terminus of Cvhsp90, which indicated it was the β-isoform of Hsp90s [35]. Two highly charged domains of CvHsp90 indicate that it more likely to bind to positively charged or hydrophobic protein and the bHLH protein folding domain suggests that CvHsp90 can rapidly convert a basic Helix-Loop-Helix protein from an inactive to an active conformation [36]–[37]. The AU-rich elements (ARE) is found located at 3′-UTR region of CvHsc70 and CvHsp90 suggested that the possible posttranscriptional regulation of them is the mRNA degradation, which is influenced by many exogenous factors, including phorbol esters, calcium ionophores, cytokines, and transcription inhibitors [38].

The role of heat shock proteins in development is less well understood, and earlier studies were only proceeding in model insects and few other insects. For examples, sHsps were continually expressed during development of *D. melanogaster* [39], expression level of Hsp70 varied among life stages of *T. castaneum* [40], and three *Hsps* increased their mRNA expression during the developmental course of *P. xylostella* [41]. In the current study, transcript abundances of four *CvHsps* were checked through each developmental stage of *C. vestali*s. We found that the transcript abundance of *CvHsp40* remained a low level during the larval stage, but increased significantly at the pupal and adult stages; the transcript abundance of *CvHsc70* remained around the same level during the larval, pupal and male adult stages, but females showed a much higher transcript abundance; the transcript abundance of *CvHsp70* is low in early and middle larval stages, and then followed by a sharp increase at later larval stage, third-instar larva; the transcript abundance of *CvHsp90* dropped at each consecutive developmental stage. The different transcriptional patterns of *CvHsps* suggested that they are under differential mechanisms. The life history of *C. vestalis* showed that the third-instar larva is a special stage [23]. At that time, *C. vestalis* larva exits the host larva and spins a cocoon outside the host, thus facing very different environment stresses. The transcriptional pattern of *CvHsp70*, which exhibited a dramatic increase at the third-instar, reveals that *CvHsp70* might be a useful biomarker to assess life history traits in future research. The gender-specific transcript increase of *CvHsp40*, *CvHsc70* and *CvHsp70* might indicate that they were required in the female reproduction of *C. vestalis* or female adult of *C. vestalis* was better at heat tolerance than male. However, it should be noted that the RNA used for the present study was extracted from the whole organism and the data obtained may reveal an average expression of *CvHsp40*, *CvHsc70* or *CvHsp70*, therefore the examination of expression of *CvHsp40*, *CvHsc70* or *CvHsp70* in different tissues and organs is apparently needed to better understand its functions.

Tolerances to extreme environmental factors, particularly temperature, can provide insight into insect biology. In insects and possibly most organisms, *Hsps* show altered expression profiles during temperature stress, particularly the maximal induction of *Hsp* transcripts. In this study, our finding that four *CvHsp* transcripts can be significantly induced by heat stress is similar to previous results [31], [35], [40]–[42]. However, the transcript abundance of *CvHsps* around 27°C is mostly significantly lower than those of other stress temperature, including 24°C, at every developmental stage, which might indicate that the temperature of 27°C is a suitable condition for development of *C. vestalis*. The tested population of *C. vestalis* was originally collected from the Hangzhou area, where this species is an abundant one in the later spring, early summer and autumn in the cruciferous vegetable area, and the average temperature in spring and autumn in this area is approximately 27°C. This might suggest that there is a possible biological relationship between the temperature at which the abundance of the *CvHsp* transcripts begins to increase and the average temperature of the distribution area of *C. vestalis*. In conclusion, (1) Four *CvHsp* genes were characterized from the endoparasitoid wasp, *C. vestalis*. (2) The divergent transcriptional patterns of *CvHsp40*, *CvHsp70 and CvHsp90* in different developmental stages suggest that *CvHsps* transcripts are under differential regulation during development. The dramatic increase of transcripts of *CvHsp70* at the third-instar larva coincided with its developmental change in this stage. (3) *CvHsp40, CvHsc70* and *CvHsp70* showed sex-specific differences of transcript abundance in the adult stage; (4) the transcripts of *CvHsps* at all developmental stages were significantly induced by heat stress; the lowest transcript abundances appeared at 27°C, which probably suggest that this is the most favorable temperature for the development of *C. vestalis*.

## Supporting Information

Figure S1 **Full length cDNA and deduced amino acid sequence of** ***CvHsp40*** **.** Asterisk indicates the translational termination codon. The putative polyadenylation signal is grey covered and dash underlined. J-domain is dash underlined. G/F domain is grey covered. C-terminal substrate binding domain is solid underlined.
(TIF)

Figure S2 **Full length cDNA and deduced amino acid sequence of** ***CvHsp70*** **.** Asterisk indicates the translational termination codon. The putative polyadenylation signal is grey covered and dash underlined. ATP-GTP binding site is dash underlined. Bipartite nuclear localization signal is solid underlined. Non-organellar consensus motif is grey covered. EEVD motif is double solid underlined.
(TIF)

Figure S3 **Full length cDNA and deduced amino acid sequence of** ***CvHsc70*** **.** Asterisk indicates the translational termination codon. The putative polyadenylation signal is grey covered and dash underlined. Two AU-rich elements (ARE) motifs are grey covered and solid underlined. ATP-GTP binding site is dash underlined. Bipartite nuclear localization signal is solid underlined. Non-organellar consensus motif is grey covered. EEVD motif is double solid underlined. Four GGMP motifs are open boxed.
(TIF)

Figure S4 **Full length cDNA and deduced amino acid sequence of** ***CvHsp90*** **.** Asterisk indicates the translational termination codon. The putative polyadenylation signal is grey covered and dash underlined. One AU-rich elements (ARE) motifs are grey covered and solid underlined. ATP-GTP binding domain is dash underlined. Charged hinge domain is grey covered. Nuclear localization signal is solid underlined. Target proteins binding domain is light grey covered. Basic Helix-Loop-Helix (bHLH) protein folding domain is open boxed. ATP-GTP binding domain is double dash underlined. EEVD motif is double solid underlined.
(TIF)

## References

[1] Shaw MR (2003) Revised synonymy in the genus *Cotesia* (Hymenoptera: Braconidae: Microgastrinae): the identity of *Microgaster vestalis* Haliday, 1834, as a senior synonym of *Apanteles plutellae* Kurdjumov, 1912. Entomol Gazette 54: 187–189.

[2] Bai SF, Chen XX, Cheng JA, Fu WJ, He JH (2003) Characterization of *Cotesia plutellae* polydnavirus and its physiological effects on the diamondback moth, *Plutella xylostella* larvae. Acta Entomol Sinica 46: 401–408.

[3] Bai SF, Chen XX, Cheng JA, Fu WJ, He JH (2005) Effects of wasp-associated factors of *Cotesia plutellae* on growth and development of *Plutella xylostella* larvae. J Plant Protect 32: 235–240.

[4] Sarfraz M, Keddie AB, Dosdall LM (2005) Biological control of the diamondback moth, *Plutella xylostella*: A review. Biocontrol Sci Technol 15: 763–789.

[5] Liu S, Wang X, Guo S, He J, Shi Z (2000) Seasonal abundance of the parasitoid complex associated with the diamondback moth, *Plutella xylostella* (Lepidoptera: Plutellidae) in Hangzhou, China. Bull Entomol Res 90: 221–231. 10996863 10.1017/s0007485300000341

[6] Shi ZH, Liu SS (1999) Influence of temperature on the development, survival and reproduction of *Cotesia plutellae*, a larval parasite of *Plutella xylostella* . Acta Phytophylacica Sinica 26: 142–146.

[7] Feder ME, Hofmann GE (1999) Heat-shock proteins, molecular chaperones, and the stress response: evolutionary and ecological physiology. Annu Rev Physiol 61: 243–282. 10099689 10.1146/annurev.physiol.61.1.243

[8] Kim KK, Kim R, Kim S (1998) Crystal structure of small heat-shock protein. Nature 394: 595–599. 9707123 10.1038/29106

[9] Sørensen JG, Kristensen GTN, Loeschcke V (2003) The evolutionary and ecological role of heat shock proteins. Ecol Lett 6: 1025–1037.

[10] Qiu XB, Shao YM, Miao S, Wang L (2006) The diversity of the DnaJ/Hsp40 family, the crucial partners for Hsp70 chaperones. Cell Mol Life Sci 63: 2560–2570. 16952052 10.1007/s00018-006-6192-6 PMC11136209

[11] Picard D (2002) Heat-shock protein 90, a chaperone for folding and regulation. Cell Mol Life Sci 59: 1640–1648. 12475174 10.1007/PL00012491 PMC11337538

[12] Asgari S, Zhang GM, Schmidt O (2003) Polydnavirus particle proteins with similarities to molecular chaperones, heat-shock protein 70 and calreticulin. J Gen Virol 84: 1165–1171. 12692281 10.1099/vir.0.19026-0

[13] Haass C, Klein U, Kloetzel PM (1990) Developmental expression of *Drosophila melanogaster* small heat-shock proteins. J Cell Sci 96: 413–418. 2121748 10.1242/jcs.96.3.413

[14] Joanisse DR, Michaud S, Inaguma Y, Tanguay RM (1998) Small heat shock proteins of *Drosophila*: developmental expression and functions. J Biosci 23: 369–376. 10.1006/bbrc.1998.8214 9514881

[15] Johnston JA, Ward CL, Kopito RR (1998) Aggresomes: a cellular response to misfolded proteins. J Cell Biol 143: 1883–1898. 9864362 10.1083/jcb.143.7.1883 PMC2175217

[16] Pockley AG (2003) Heat shock proteins as regulators of the immune response. Lancet 362: 469–476. 12927437 10.1016/S0140-6736(03)14075-5

[17] Rinehart JP, Denlinger DL, Rivers DB (2002) Upregulation of transcripts encoding select heat shock proteins in the flesh fly *Sarcophaga crassipalpis* in response to venom from the ectoparasitoid wasp *Nasonia vitripennis* . J Invertebr Pathol 79: 62–63. 12054789 10.1016/S0022-2011(02)00002-2

[18] Rinehart JP, Li A, Yocum GD, Robich RM, Hayward SA, Denlinger DL (2007) Up-regulation of heat shock proteins is essential for cold survival during insect diapauses. Proc Natl Acad Sci USA 104(27): 11130–11137. 17522254 10.1073/pnas.0703538104 PMC2040864

[19] Shim JK, Ha DM, Nho SK, Song KS, Lee KY (2008) Upregulation of heat shock protein genes by envenomation of ectoparasitoid *Bracon hebetor* in larval host of Indian meal moth *Plodia interpunctella* . J Invertebr Pathol 97: 306–309. 17981295 10.1016/j.jip.2007.10.001

[20] Zhu JY, Fang Q, Wang L, Hu C, Ye GY (2010) Proteomic analysis of the venom from the endoparasitoid wasp *Pteromalus Puparum* (Hymenoptera: Pteromalidae). Arch Insect Biochem Physiol 75: 28–44. 20648599 10.1002/arch.20380

[21] Rutherford SL (2003) Between genotype and phenotype: Protein chaperones and evolvability. Nat Rev Genet 4: 263–274. 12671657 10.1038/nrg1041

[22] Sørensen JG, Loeschcke V (2001) Larval crowding in *Drosophila melanogaster* induces Hsp70 expression, and leads to increased adult longevity and adult thermal stress resistance. J Insect Physiol 47: 1301–1307. 12770182 10.1016/s0022-1910(01)00119-6

[23] Yu RX, Shi M, Huang F, Chen XX (2008) Immature Development of *Cotesia vestalis* (Hymenoptera: Braconidae), an Endoparasitoid of *Plutella xylostella* (Lepidoptera: Plutellidae). Ann Entomol Soc Am 101(1): 189–196.

[24] Thompson JD, Gibson TJ, Plewniak F, Jeanmougin F, Higgins DG (1997) The CLUSTAL_X windows interface: flexible strategies for multiple sequence alignment aided by quality analysis tools. Nucleic Acids Res 25(24): 4876–4882. 9396791 10.1093/nar/25.24.4876 PMC147148

[25] Kumar S, Nei M, Dudley J, Tamura K (2008) MEGA: a biologist-centric software for evolutionary analysis of DNA and protein sequences. Brief Bioinform 9(4): 299–306. 18417537 10.1093/bib/bbn017 PMC2562624

[26] Felsenstein J (1985) Confidence limits on phylogenies: an approach using the bootstrap. Evolution 39: 783–791. 28561359 10.1111/j.1558-5646.1985.tb00420.x

[27] Peirson SN, Butler JN, Foster RG (2003) Experimental validation of novel and conventional approaches to quantitative real-time PCR data analysis. Nucleic Acids Res 31: e73. 12853650 10.1093/nar/gng073 PMC167648

[28] Livak KJ, Schmittgen TD (2001) Analysis of relative gene expression data using real-time quantitayive PCR and the 2T-Delta Delta C method. Methids 25: 402–408. 10.1006/meth.2001.1262 11846609

[29] Caplan AJ, Cyr DM, Douglas MG (1993) Eukaryotic homologs of *Escherichia coli* DnaJ: a diverse protein family that functions with Hsp70 stress proteins. Mol Biol Celt 4: 555–563. 10.1091/mbc.4.6.555 PMC300962 8374166

[30] Fan CY, Lee S, Ren HY, Cyr DM (2004) Exchangeable chaperone modules contribute to specification of type I and type II Hsp40 cellular function. Mol Biol Cell 15: 761–773. 14657253 10.1091/mbc.E03-03-0146 PMC329391

[31] Zhang QR, Denlinger DL (2010) Molecular characterization of heat shock protein 90, 70 and 70cognate cDNAs and their expression patterns during thermal stress and pupal diapause in the corn earworm. J Insect Physiol 56: 138–150. 19782689 10.1016/j.jinsphys.2009.09.013

[32] Gupta RS (1995) Phylogenetic analysis of the 90 kD heat-shock family of protein sequences and an examination of the relationship among animals, plants, and fungi species. Mol Biol Evol 12: 1063–1073. 8524040 10.1093/oxfordjournals.molbev.a040281

[33] Vayssier M, Le Guerhier F, Fabien JF, Philippe H, Vallet C, Ortega-Pierres G, Soule C, Perret C, Liu M, Vega-Lopez M, Boireau P (1999) Cloning and analysis of a *Trichinella britovi* gene encoding a cytoplasmic heat shock protein of 72 kDa. Parasitology 119: 81–93. 10446707 10.1017/s0031182099004461

[34] Demand J, Lüders J, Höhfeld J (1998) The carboxy-terminal domain of HSC70 provides binding sites for a distinct set of chaperone cofactors. Mol Cell Biol 18: 2023–2028. 9528774 10.1128/mcb.18.4.2023 PMC121432

[35] Gao Q, Zhao J, Song L, Qiu L, Yu Y, Zhang H, Ni D (2008) Molecular cloning, characterization and expression of heat shock protein 90 gene in the haemocytes of bay scallop *Argopecten irradians* . Fish Shellfish Immunol 24: 379–385. 18282767 10.1016/j.fsi.2007.08.008

[36] Csermely P, Schnaider T, Soti C, Prohászka Z, Nardai G (1998) The 90-kDa molecular chaperone family: structure, function, and clinical applications. A comprehensive review. Pharmacol Ther 79(2): 129–68. 9749880 10.1016/s0163-7258(98)00013-8

[37] Shaknovich R, Shue G, Kohtz DS (1992) Conformational activation of a basic helix-loop-helix protein (MyoD1) by the C-terminal region of murine HSP90 (HSP84). Mol Cell Biol 12(11): 5059–5068. 1406681 10.1128/mcb.12.11.5059-5068.1992 PMC360439

[38] Chen CYA, Shyu AB (1995) AU-rich elements: characterization and importance in mRNA degradation. Trends Biochem Sci 20 (11): 465–470. 10.1016/s0968-0004(00)89102-1 8578590

[39] Kurzik-Dumke U, Lohmann E (1995) Sequence of the new Drosophila melanogaster small heat-shock-related gene, lethal (2) essential for life [l(2)efl], at locus 59F4,5. Gene 154(2): 171–175. 7890160 10.1016/0378-1119(94)00827-f

[40] Mahroof R, Zhu KY, Subramanyam B (2005) Changes in Expression of Heat Shock Proteins in *Tribolium castaneum* (Coleoptera: Tenebrionidae) in Relation to Developmental Stage, Exposure Time, and Temperature. Ann Entomol Soc Am 98(1): 100–107.

[41] Sonoda S, Ashfaq M, Tsumuki H (2006) Cloning and nucleotide sequencing of three heat shock protein genes (hsp90, hsc70, and hsp19.5) from the diamondback moth, *Plutella xylostella* (L.) and their expression in relation to developmental stage and temperature. Arch Insect Biochem Physiol 62: 80–90. 16703614 10.1002/arch.20124

[42] Wang H, Li K, Zhu JY, Fang Q, Ye GY (2012) Cloning and expression pattern of heat shock protein genes from the endoparasitoid wasp, *Pteromalus puparum* in response to environmental stresses. Arch Insect Biochem Physiol 79(4–5): 247–263. 22517445 10.1002/arch.21013

