# Supplementary figures and images for: Four Heat Shock Protein Genes of the Endoparasitoid Wasp, Cotesia vestalis, and Their Transcriptional Profiles in Relation to Developmental Stages and Temperature
Source: PLoS One. 2013 Mar 18;8(3):e59721. doi: 10.1371/journal.pone.0059721 (PMC3601058; doi:10.1371/journal.pone.0059721)

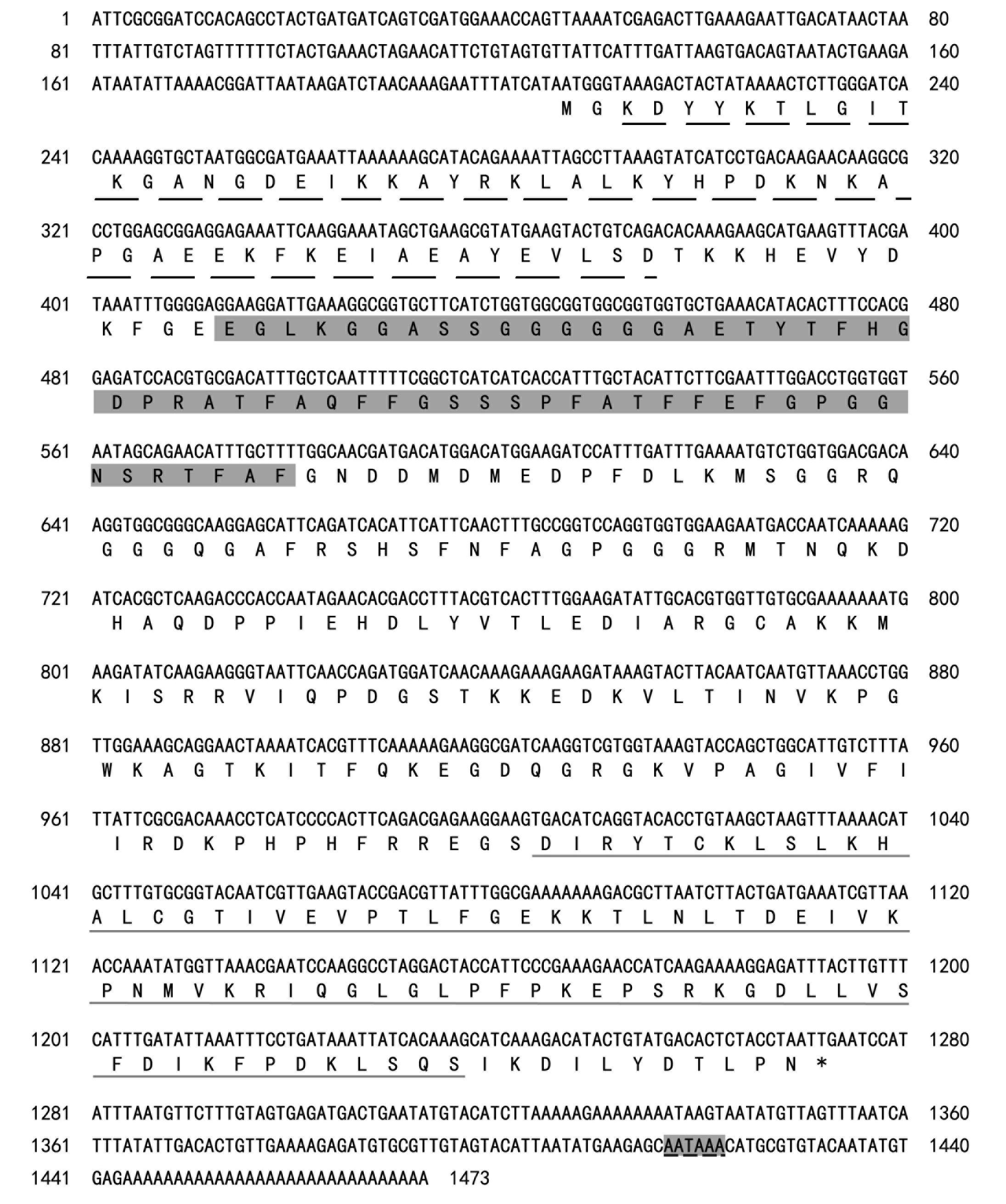

Supplement: Figure S1 — Full length cDNA and deduced amino acid sequence of CvHsp40 . Asterisk indicates the translational termination codon. The putative polyadenylation signal is grey covered and dash underlined. J-domain is dash underlined. G/F domain is grey covered. C-terminal substrate binding domain is solid underlined. (TIF) [file pone.0059721.s001.tif]

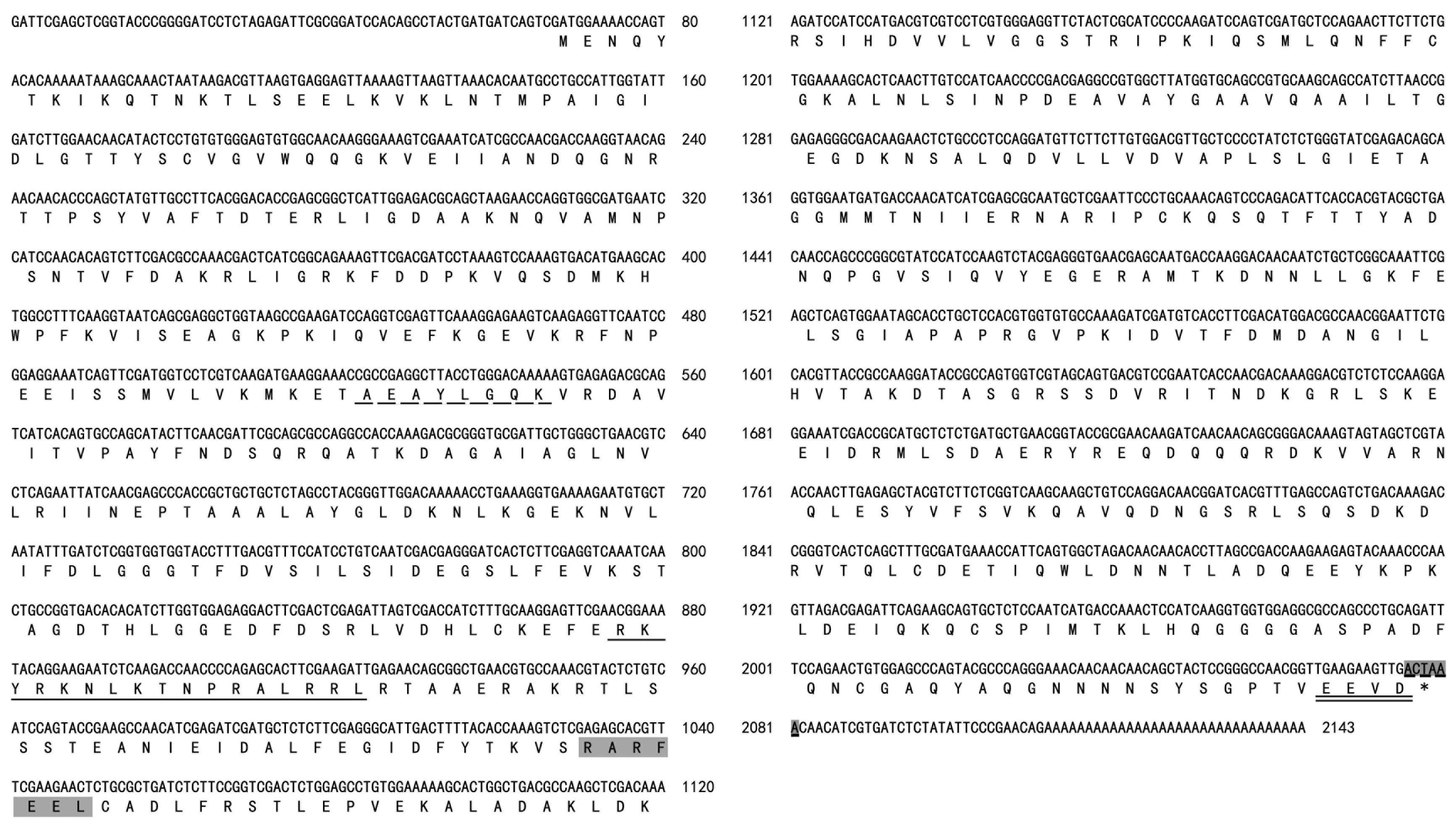

Supplement: Figure S2 — Full length cDNA and deduced amino acid sequence of CvHsp70 . Asterisk indicates the translational termination codon. The putative polyadenylation signal is grey covered and dash underlined. ATP-GTP binding site is dash underlined. Bipartite nuclear localization signal is solid underlined. Non-organellar consensus motif is grey covered. EEVD motif is double solid underlined. (TIF) [file pone.0059721.s002.tif]

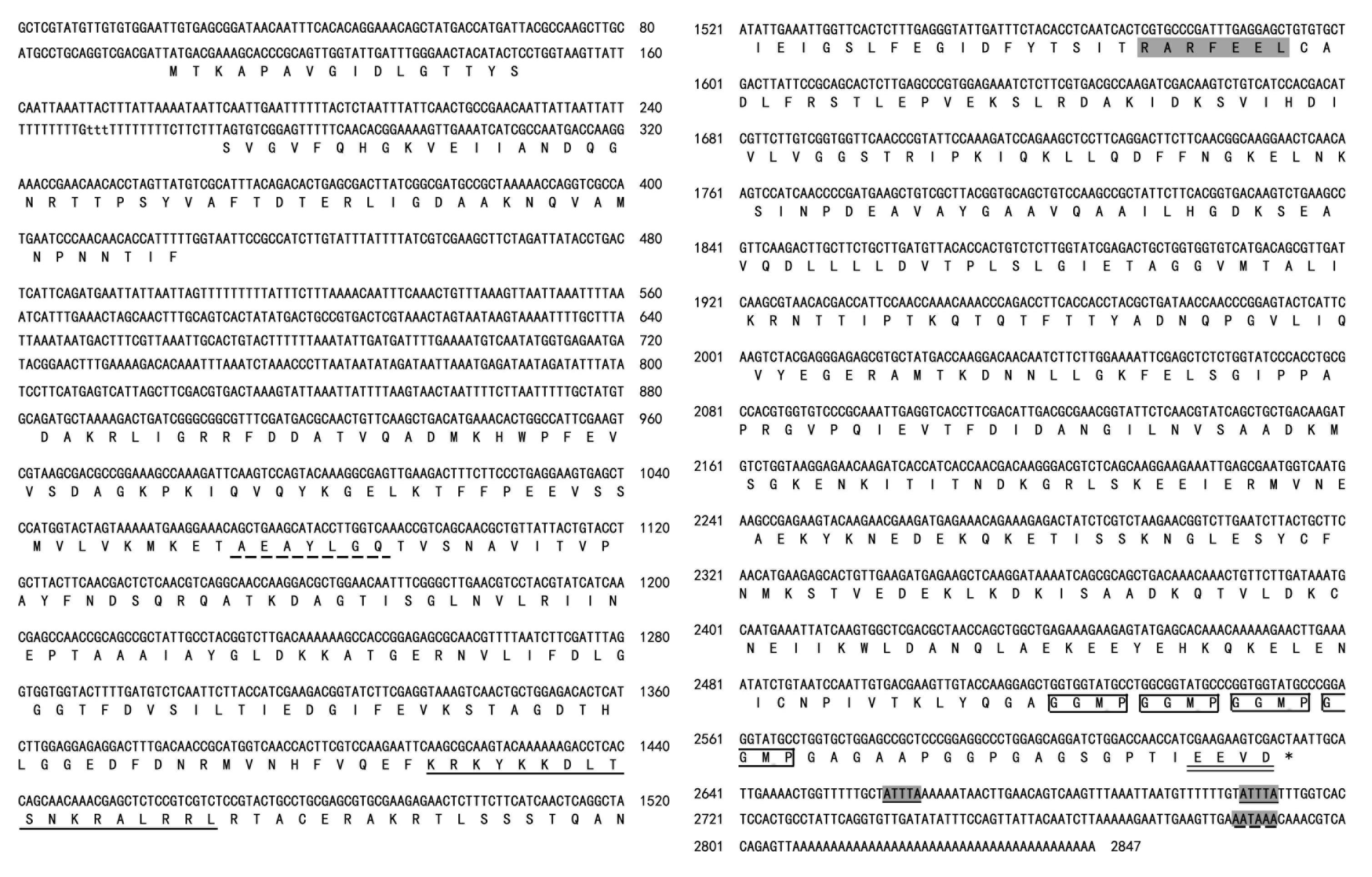

Supplement: Figure S3 — Full length cDNA and deduced amino acid sequence of CvHsc70 . Asterisk indicates the translational termination codon. The putative polyadenylation signal is grey covered and dash underlined. Two AU-rich elements (ARE) motifs are grey covered and solid underlined. ATP-GTP binding site is dash underlined. Bipartite nuclear localization signal is solid underlined. Non-organellar consensus motif is grey covered. EEVD motif is double solid underlined. Four GGMP motifs are open boxed. (TIF) [file pone.0059721.s003.tif]

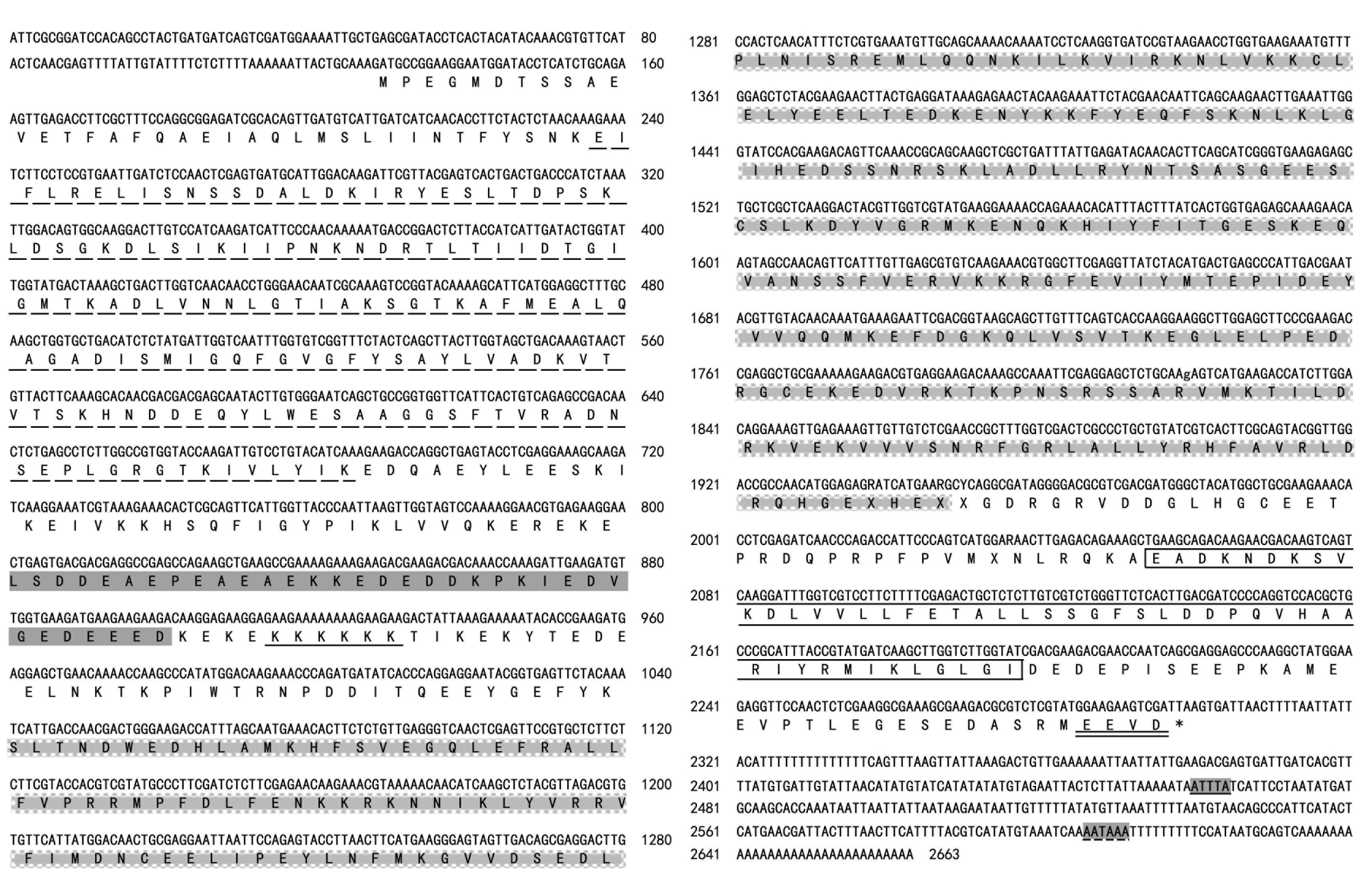

Supplement: Figure S4 — Full length cDNA and deduced amino acid sequence of CvHsp90 . Asterisk indicates the translational termination codon. The putative polyadenylation signal is grey covered and dash underlined. One AU-rich elements (ARE) motifs are grey covered and solid underlined. ATP-GTP binding domain is dash underlined. Charged hinge domain is grey covered. Nuclear localization signal is solid underlined. Target proteins binding domain is light grey covered. Basic Helix-Loop-Helix (bHLH) protein folding domain is open boxed. ATP-GTP binding domain is double dash underlined. EEVD motif is double solid underlined. (TIF) [file pone.0059721.s004.tif]
